# Increased Susceptibility to Mechanical Stretch Drives the Persistence of Keloid Fibroblasts: An Investigation Using a Stretchable PDMS Platform

**DOI:** 10.3390/biomedicines12102169

**Published:** 2024-09-24

**Authors:** Jihee Kim, Chihyeong Won, Seoyoon Ham, Heetak Han, Sungsik Shin, Jieun Jang, Sanghyeon Lee, Chaebeen Kwon, Sungjoon Cho, Hyeonjoo Park, Dongwon Lee, Won Jai Lee, Taeyoon Lee, Ju Hee Lee

**Affiliations:** 1 Department of Dermatology, Cutaneous Biology Research Institute, Yonsei University College of Medicine, Seoul 03722, Republic of Korea; mygirljihee@yuhs.ac (J.K.); hsy7852@gmail.com (S.H.); sungsik@yuhs.ac (S.S.); jieun@yuhs.ac (J.J.); 2 School of Electrical and Electronic Engineering, Yonsei University, Seoul 03722, Republic of Korea; chihyeong.won14@gmail.com (C.W.); kamacoon@yonsei.ac.kr (H.H.); sha1140@yonsei.ac.kr (S.L.); ccoun012@gmail.com (C.K.); sungjoon.cho97@gmail.com (S.C.); hj01@yonsei.ac.kr (H.P.); 3 Andrew and Peggy Cherng Department of Medical Engineering, Division of Engineering and Applied Science, California Institute of Technology, Pasadena, CA 91125, USA; 4 Department of Plastic and Reconstructive Surgery, Institute for Human Tissue Restoration, Yonsei University College of Medicine, Seoul 03722, Republic of Korea; xyphoss@yuhs.ac (D.L.); pswjlee@yuhs.ac (W.J.L.)

**Keywords:** keloid, fibroblast, mechanosensitivity, polydimethylsiloxane

## Abstract

Background: Keloids are a common fibrotic disease of the skin, with the pathological hallmark of excessive extracellular matrix synthesis due to abnormal fibroblast activity. Since keloids clinically arise in areas of high mechanical tension, the mechanotransductory pathway may be attributed to its pathogenesis. We aimed to establish a preclinical platform to elucidate the underlying mechanism of keloid development and its clinical persistence. Methods: We fabricated a mechanically stretchable polydimethylsiloxane cell culture platform; with its mimicry of the in vivo cyclic stretch of skeletal muscles, cells showed higher proliferation compared with conventional modalities. Results: In response to mechanical strain, TGF-β and type 1 collagen showed significant increases, suggesting possible TGF-β/Smad pathway activation via mechanical stimulation. Protein candidates selected by proteomic analysis were evaluated, indicating that key molecules involved in cell signaling and oxidative stress were significantly altered. Additionally, the cytoskeletal network of keloid fibroblasts showed increased expression of its components after periodic mechanical stimulation. Conclusions: Herein, we demonstrated and validated the existing body of knowledge regarding profibrotic mechanotransduction signaling pathways in keloid fibroblasts. Cyclic stretch, as a driving force, could help to decipher the tension-mediated biomechanical processes, leading to the development of optimized therapeutic targets.

## 1. Introduction

Fibrosis refers to the process of abnormal or excessive accumulation of extracellular matrix (ECM) substrates, the key drivers of progressive organ dysfunction in many diseases [1,2]. It is understood that acute and chronic injury of the skin can provoke a fibrotic process, leading to aberrations in the physiological wound-healing process [3]; however, the mechanism responsible for persistence of this response is not well understood. Abnormalities in mechanical characteristics have been noted in both the cells and ECM structure during the systemic fibrotic process [4]; current genetic analysis revealed the aberrant expression of genes involved in tissue remodeling in response to abnormal tensile force [5,6]. Hypertrophic scars and keloids are common fibroproliferative diseases, characterized by the histopathological hallmark of excessive ECM synthesis due to abnormal fibroblast activity [7,8]. Although benign, keloids may cause pruritus, pain, and even contractures, which significantly affect the patients’ quality of life, both physically and psychologically. To date, numerous hypotheses have been proposed to explain the underlying mechanisms of keloid formation including genetic predisposition [9,10], upregulation of growth factors and inflammatory cytokines [11,12], and regulation of cellular apoptosis [13].

Transforming growth factor-beta (TGF-β), a crucial mediator in keloid pathogenesis [14,15], as well as myofibroblast activity, are the two central elements linked to mechanotransduction during fibrotic processes [6,16]. Mechanical stimuli can trigger a variety of cellular responses, including alterations in cell morphology, cell function, and gene expression [17,18]. An increase in mechanical tension induces myofibroblast proliferation and ECM accumulation [5,19]. Previous studies demonstrated that tension-regulated mechanical stress induces the interplay between fibronectin and collagen fibers during ECM assembly in the normal wound-healing process [20]. Clinically, keloids tend to develop in tension-prone sites like the chest, back, shoulders, and lower abdomen due to frequent stretching from body movements. They tend to grow both vertically and horizontally, with the direction of horizontal growth varying by location. This variation leads to distinct shapes that reflect the primary directions of skin tension [19,21,22,23]. Additionally, mechanical forces on keloid fibroblasts increase the expression of tension-related proteins and ECM components, further promoting keloid formation [5,23,24]. Additionally, the cellular characteristics of fibroblasts are altered under mechanical stress, inducing increased proliferation and decreased apoptosis. Nevertheless, quantitative studies regarding the association between mechanical stimuli and keloid development are insufficient due to the limitations of in vivo experimental models, as well as the lack of biomechanical tools. Keloids have limited treatment options and show persistence scars with conventional treatment [25,26]; however, current experimental platforms rely on the use of conventional two-dimensional (2D) cell cultures, which are proven to be poor representatives of native physiology. To date, few in vitro platforms have been fabricated to investigate cell responses under the influence of mechanical stimuli [27]. Strain devices with expandable silicone (such as polydimethylsiloxane (PDMS)) culture membranes are frequently used to stretch cells in vitro, thereby mimicking mechanically dynamic tissue environments [28,29]. Based on these studies, in this study, we developed a coupled, mechanically stretchable cell culture platform set that generates a cyclic stretch, effectively mimicking physiological conditions. This platform was then used to elucidate the key mechanosensitive pathways that induce fibrogenic properties, which would allow for the development of new, knowledge-based, preventative, and therapeutic treatments.

## 2. Materials and Methods

### 2.1. Keloid Tissues and Human Dermal Fibroblasts

This study protocol was approved by the Yonsei University College of Medicine Institutional Review Board (IRB No. 4-2015-0228). All experiments involving humans were performed in accordance with the Declaration of Helsinki guidelines, and written informed consent was provided by all patients. Human keloid tissue samples were obtained from active-stage keloid patients (*n* = 5). The patient information has been provided in the Supplementary Table S1. Primary cultures of KFs were obtained by growing the cells on culture dishes (SPL) in Dulbecco’s modified Eagle’s medium (Lonza Group, Basel, Switzerland) supplemented with 10% fetal bovine serum, 100 U/mL penicillin, and 0.1 mg/mL streptomycin at 37 °C and 5% CO_2_. The culture medium was changed at 2–3-day intervals, and cells were used for experiment after 2~3 rotations. HDFs were purchased from Thermo Fisher Scientific (Carlsbad, CA, USA) and cultured for 6–9 passages in RPMI 1640 medium (Lonza). Afterwards, both cell cultures were transferred onto a PDMS plate at a density of 2 × 10^4^ cells/well in 0.5 mL culture medium.

### 2.2. Cell Proliferation Assay

HDFs and KFs were seeded in PDMS plates at a concentration of 2 × 10^4^ cells/well for 24 h. PDMS plates were attached to a mechanical stretch by a computer-controlled step motor. The fibroblasts were exposed to mechanical tension for 1 h per day for the initial 3 days of the culture period, and then further cultured for 7 days. The proliferation assay results were analyzed using the Cell Counting Kit-8 (Dojindo Laboratories, Kumamoto, Japan).

### 2.3. Gene Expression Profile

Total RNA was extracted using the RNeasy Plus Mini Kit (Qiagen, Hilden, Germany) according to the manufacturer’s instructions. Equal amounts of RNA (1 μg) were converted to cDNA using TaqMan Mastermix (Applied Biosystems, Foster City, CA, USA) and a real-time PCR system (Applied Biosystems). The expression levels were evaluated for the following genes: fibronectin (Hs01549976_m1), COL1A1 (Hs00164004_m1), and TGF-β (Hs00998133_m1); expression levels were normalized to those of GAPDH (Hs02758991_g1). Relative quantification was performed using Applied Biosystems 7500 software version 2.0.1.

### 2.4. Quantitative Proteomics Analysis Using a TMT-Labelling Method

Mixtures of proteins from each group of stretching cells were reduced with 500 mM Tris (2-carboxyethyl) phosphine for 60 min, and subsequently alkylated with 500 mM iodoacetamide (IAA) for 60 min in the dark. The samples were desalted using 10-kDa membrane filters and dissolved in 100 μL of 200 mM triethylammoniumbicarbonate (TEAB). Protein content was analyzed using a bicinchoninic acid assay (Sigma, St. Louis, MO, USA). Sequencing-grade trypsin (Promega, Madison, WI, USA) was added at an enzyme-to-protein ratio of 1:20 (*w*/*w*) in TEAB buffer, and incubated overnight at 37 °C. Samples were labelled using TMT-126, -128, and -130 (stretched group), or TMT-127, -129, and 131 (control group), according to the manufacturer’s protocol (Thermo Scientific, Waltham, MA, USA). An aqueous hydroxylamine solution (5% *w*/*v*) was added to quench the reaction. The six samples were then combined, speed vacuum-dried, and redissolved in 50 μL of water containing 0.1% formic acid for analysis using 2D-liquid chromatography-tandem mass spectrometry.

### 2.5. Western Blotting 

HDFs and KFs were washed with phosphate-buffered saline (PBS), collected with trypsin, and centrifuged. The cells were lysed in cold RIPA buffer with a protease inhibitor cocktail tablet. Protein content was analyzed using the bicinchoninic acid solution (Sigma); equal amounts of protein were separated by 10–12% sodium dodecyl sulphate-polyacrylamide gel electrophoresis and transferred to nitrocellulose blotting membranes (GE Healthcare, Amersham, Buckinghamshire, UK). The transferred membranes were blocked for 1 h with 5% skim milk in Tris-buffered saline with Tween 20 (TBST), and incubated overnight on a shaker at 4 °C with rabbit anti-PPP2R5D (1:500, Abcam, Cambridge, UK), rabbit anti-PDLIM5 (1:500, Abcam), rabbit anti-AKR1B1 (1:500, Abcam), mouse SOD-2 (1:500, Santa Cruz Biotechnology, Dallas, TX, USA), rabbit anti-TGF-β (1:200, Abcam), rabbit anti-COL1A1 (1:200, Santa Cruz Biotechnology), and mouse anti-β-actin (1:1000, Santa Cruz Biotechnology) antibodies. The membranes were washed with TBST and incubated for 1 h with opposite horseradish peroxidase-conjugated secondary antibodies (1:2000, Cell Signalling, Danvers, MA, USA). 

### 2.6. Immunofluorescent Staining

HDFs and KFs were fixed in 4% paraformaldehyde after exposure to mechanical tension. OCT-embedded normal and keloid tissues were sectioned, and the slides were fixed in 4% paraformaldehyde. The cells and tissues were washed with PBS for 15 min, and permeabilized with 0.25% Tween 20 in Tris-buffered saline three times; the slides were then blocked with 5% bovine serum albumin in TBST for 1 h. The cells were incubated overnight in a cold room with the following primary antibodies: rabbit anti-AFAP (1:200, Novus Biologicals, Centennial, CO, USA), mouse anti-fibronectin (1:200, Abcam), rabbit anti-α-tubulin (1:2000, Abcam), mouse anti-vimentin (1:2000, Abcam), rabbit anti-PPP2R5D (1:200, Abcam), mouse anti-PDLIM5 (1:200, Abcam), rabbit anti-AKR1B1 (1:200, Abcam), mouse SOD-2 (1:200, Santa Cruz Biotechnology), rabbit anti-TGF-β (1:200, Abcam), and mouse anti-COL1A1 (1:200, Santa Cruz Biotechnology) antibodies. Secondary antibodies included fluorescein isothiocyanate-conjugated anti-mouse, and Alexa 555-conjugated anti-rabbit (Invitrogen, Cergy Pontoise, France). The slides were washed with TBST, mounted with mounting media with DAPI (Vector Laboratories, Burlingame, CA, USA), and examined using a confocal microscope.

### 2.7. Statistical Analysis

Statistical analysis was performed using the two-tailed Student’s *t*-test for two-group comparison; the results are expressed as the mean ± standard deviation (SD). Consecutive and sequential data were analyzed by repeated one-way analysis of variance (ANOVA). Differences were considered statistically significant when *p* < 0.05.

## 3. Results

### 3.1. Fabrication of a High-Throughput, Mechanically Stretchable PDMS Cell Culture Platform

**Figure 1:**
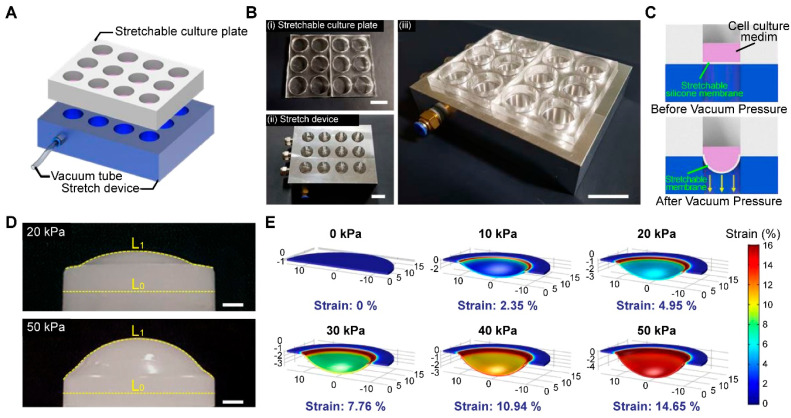
Fabrication of the high-throughput, mechanically stretchable, PDMS cell culture platform (**A**) A static overview of the mechanically stretchable cell culture platform; (**B**) the PDMS platform consisted of 12-wells (**iii**), with two major components: a stretchable culture plate (**i**), and a stretch device controlled by vacuum pressure (**ii**) (scale bar: 20 mm); (**C**) mechanical stimulation was provided by adjusting the pneumatic pressure; (**D**) fabrication of the replica modeling on the stretchable platform; initial length (L0) and stretch length (L1) were compared (scale bar: 4 mm); (**E**) finite-element method to compare the applied pneumatic pressure to the mechanical strain of the PDMS platform.

A static diagram of the mechanically stretchable cell culture platform is illustrated in Figure 1A. The platform consists of two major components: a stretchable culture plate, and a stretch device controlled by vacuum pressure (Figure 1B). PDMS, a well-known biocompatible elastomer, was used as the base material of the stretchable culture plates, designed to closely match the dimensions of a standard 12-well plate. The use of a PDMS-based culture system facilitates the direct visualization of the cells of interest, owing to its high optical transparency. A thin layer (500 μm) of the PDMS membrane was fabricated by drop-casting the PDMS solution onto a predesigned aluminum mold bonded to a 12-well drilled support. The stretch device was made of aluminum and was efficiently designed to simultaneously insufflate air from the uppermost layer to the bottom in multi-wells by connecting a single vacuum tube. The specific dimensions of the PDMS platform are mentioned in the Supplementary Figure S1. Mechanical stimulation was provided by adjusting the pneumatic pressure on the PDMS platform as illustrated in Figure 1C. Since the target cells adhere to the lowermost portion of the substrate during cell culture, the mechanical stimulation regulated by pneumatic stress is directly transmitted to the stretchable cell culture plate. The PDMS platform functionally adapts its form by slacking downward with increased pressure. The mechanical strain was quantitatively measured by comparing the initial length (L0) and stretched length (L1) (Figure 1D). The degree of applied pneumatic pressure was adjusted by regulating the force delivered to the stretchable cell culture plate. To verify the correlation between the applied pneumatic pressure and mechanical stretch, computational modeling was performed using the finite-element method. Before applying vacuum pressure, the cell culture plate showed negligible strain (~0.19%) due to the moderate elastic modulus of PDMS (~2 MPa) that can sustain the pressure of 1 mL of the cell culture medium (~31 Pa); after applying vacuum pressure, a significant pressure alteration was measured across the PDMS substrate. The simulated strain on the stretchable cell culture plate was consistent with experimentally observed results (Supplementary Figure S3A). The mechanical strain was exhibited with low response time (~0.2 s) followed by the repeated pneumatic pressure with a frequency of 1 Hz (Supplementary Figure S3B). In addition, the platform showed stable performance without noticeable degradation after 24 h of operation (Supplementary Figure S3C).

### 3.2. Human Dermal Fibroblasts (HDFs) and Keloid Fibroblasts (KFs) Showed Increased Proliferation Rates and Morphological Changes in Response to Mechanical Strain

HDFs and KFs were cultured on the fabricated PDMS platform; both groups of cells underwent mechanical stretch, induced by pneumatic pressure at increasing degrees. For further evaluation, the degree of mechanical stretch generated by pneumatic pressure was set to round numbers based on the previously obtained data. To induce a physiologically mimicking milieu by referring to previous papers [30,31], mechanical pressure was applied for 1 h per day at 37 °C and 5% CO_2_ for up to 7 days; we measured cell proliferation on days 1, 3, and 7 using the WST-1 assay (Supplementary Figure S4). 

**Figure 2:**
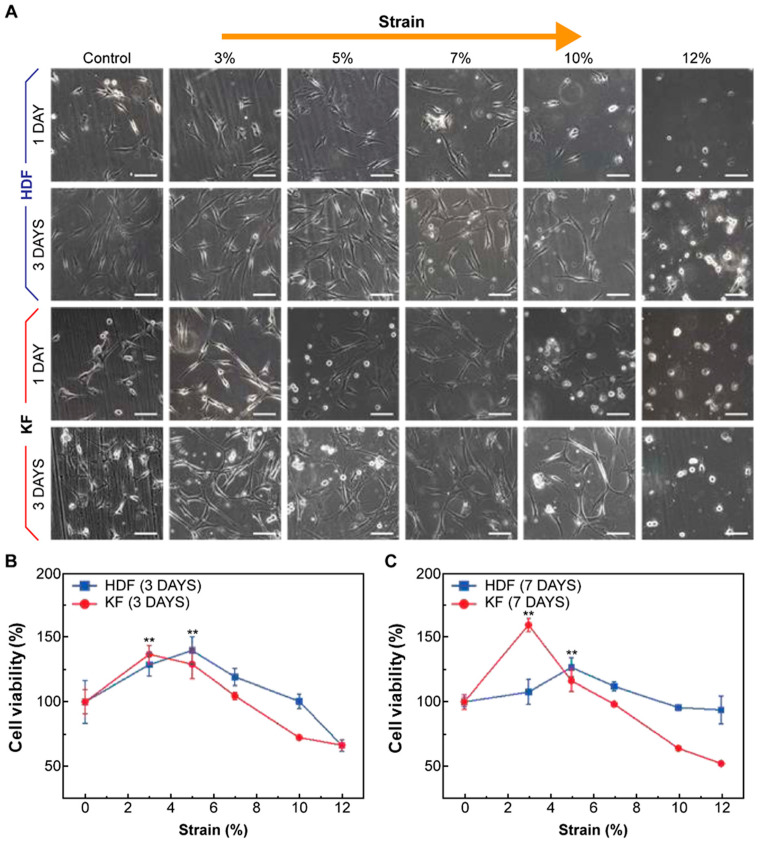
Characterization of HDFs and KFs in response to cyclic mechanical stretch. (**A**) HDFs and KFs demonstrated increased proliferation rates, as well as morphological changes, in response to mechanical strain. Cell morphology and proliferation of HDFs and KFs on the PDMS plate were analyzed via MTT assay after mechanical tension. The images were captured using a light microscope, and the scale bar represents 100 μm. Proliferation of KFs was observed at a lower degree of mechanical strain (3%) than that of HDFs (5%) after three (**B**) and seven days (**C**) of cell culture. We used HDF at passage 6~9 and KF at passage 6. All experiments were repeated three times. Error bars, SD ** *p* < 0.005; Student’s *t*-test.

Mechanical strain loaded onto the HDFs and KFs induced a significant increase in cell proliferation, corresponding with the heightened degree of applied strain (Figure 2A). Upon undergoing mechanical stretch, both types of fibroblasts became thicker, showing a more elongated morphology. Unlike the regular, parallel pattern observed in a wound-healing assay model, mechanical force induced the haphazard aggregation of HDFs. Notably, the maximum proliferation of KFs was observed at a lower degree of mechanical strain (3%) than HDFs (5%) (** *p* < 0.005; Figure 2B). The morphology under mechanical strain for HDFs and KFs is presented in the Supplementary Material (Supplementary Figure S5A,B).

### 3.3. Mechanical Stretch Induced the Expression of Fibrotic Markers and ECM Components in HDFs and KFs 

**Figure 3:**
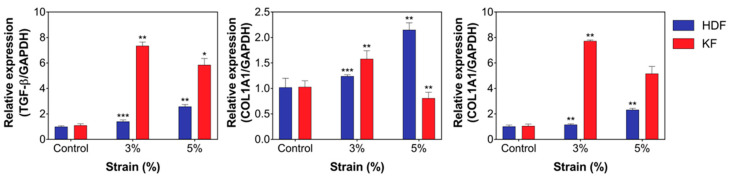
Mechanical stretch induced the increased expression of fibrotic markers and ECM components in HDFs and KFs. Gene expression profiles of TGF-β signaling and subsequent ECM accumulation on the PDMS plate after mechanical tension of RT-PCR. Mechanical tension induced increased expression of TGF-β and COL1A1 in HDFs and KFs. The expression of molecular markers was more significant with lower degrees of mechanical strain (3%) in KFs compared with HDFs (5%). We used HDFs at passage 6~9 and KFs at passage 6. All experiments were repeated three times. Error bars, SD. *** *p* < 0.001, ** *p* < 0.005, * *p* < 0.05; Student’s *t*-test.

Next, we evaluated whether mechanical stretch-induced fibroblast activation was involved in the activation of TGF-β signaling and subsequent ECM accumulation. Changes in the expression levels of profibrogenic TGF-β, type 1 collagen (COL1A1), and fibronectin were measured by RT-PCR; a significant increase in TGF-β, COL1A1, and fibronectin levels was noted in both groups of cells (** *p* < 0.005, * *p* < 0.05; Figure 3). The most noteworthy result is that cyclic mechanical stretch promoted a concurrent increase in collagen and fibronectin levels; this can be further interpreted as a clue towards explaining the persistence of the disease following its initiation. Results of the protein expression analysis were in line with those of the cell proliferation assay, in which KFs were characterized by a lower threshold for mechanical strain. Since our results demonstrated that KFs exhibit maximum proliferation and fibrotic marker expression at a lower degree of mechanical strain (3%) than HDFs (5%), we used the same condition for further in-depth experiments. 

### 3.4. Quantitative Proteomic Analysis Using a Tandem Mass Tag (TMT)-Labelling Method Revealed Potential Target Proteins with Increased Mechanosensitivity in KFs 

**Figure 4:**
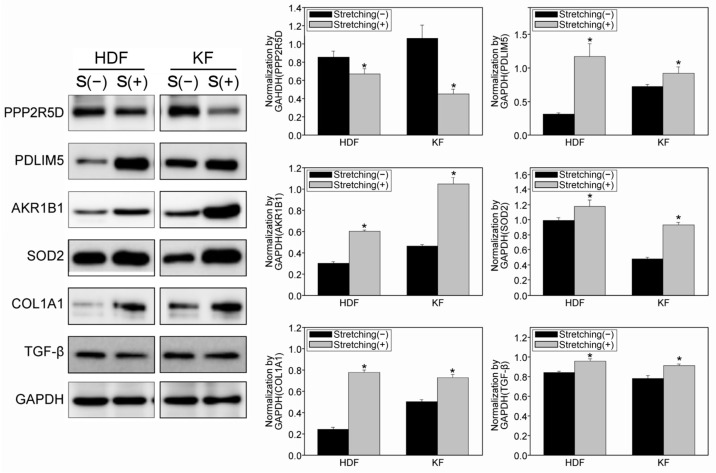
Quantitative proteomic analysis using the TMT-labeling method demonstrated target proteins subject to increased mechanosensitivity in KFs. The degree of mechanical strain was 3% for KFs and 5% for HDFs. Western blot protein expression profiles of the cell proliferation (PPP2R5D, PDLIM5), ROS (AKR1B1, SOD2), and fibrosis (COL1A1, TGF-β) markers on the PDMS plate after mechanical tension. Quantitative protein analysis identified the target proteins subject to increased mechanosensitivity in KFs. The original image of the Western blot is in Figure S6. We used HDFs at passage 6~9 and KFs at passage 6. All experiments were repeated three times. Error bars, s.e.m. * *p* < 0.05; Student’s *t*-test. Western blot images are cropped for serial comparison between each molecule.

Proteomic analysis was performed to substantiate the underlying molecular mechanism of the increased sensitivity to mechanical stress in KFs. Twenty-seven candidate protein spots were selected and analyzed via TMT labelling. In response to mechanical stretch, HDFs demonstrated 16 upregulated, and 11 downregulated proteins; in brief, the differences were most notable for proteins related to apoptosis, cellular metabolism, and reactive oxygen species (ROS) metabolism (Supplementary Tables S2 and S3). The difference in expression of candidate proteins was evaluated in response to mechanical stretch in HDFs and KFs; the cells were subject to mechanical strain to induce sufficient proliferation as described in previous results (Figure 2). PPP2R5D and PDLIM5, markers for cell proliferation and growth, showed a significant decrease and increase, respectively, in expression levels when exposed to cyclic stretch; conversely, the levels of AKR1B1 and SOD2 involved in the ROS pathway, were significantly increased (** *p* < 0.005, * *p* < 0.05; Figure 4 and Figure S6). Overall, the proteomic analysis and Western blot results suggest that abnormal fibroblast activity may be promoted by cyclic mechanical strain. 

### 3.5. Immunohistochemical Staining of KFs Demonstrated an Increase and Change in the Cytoskeletal Composition Due to Cyclic Mechanical Stretch 

**Figure 5:**
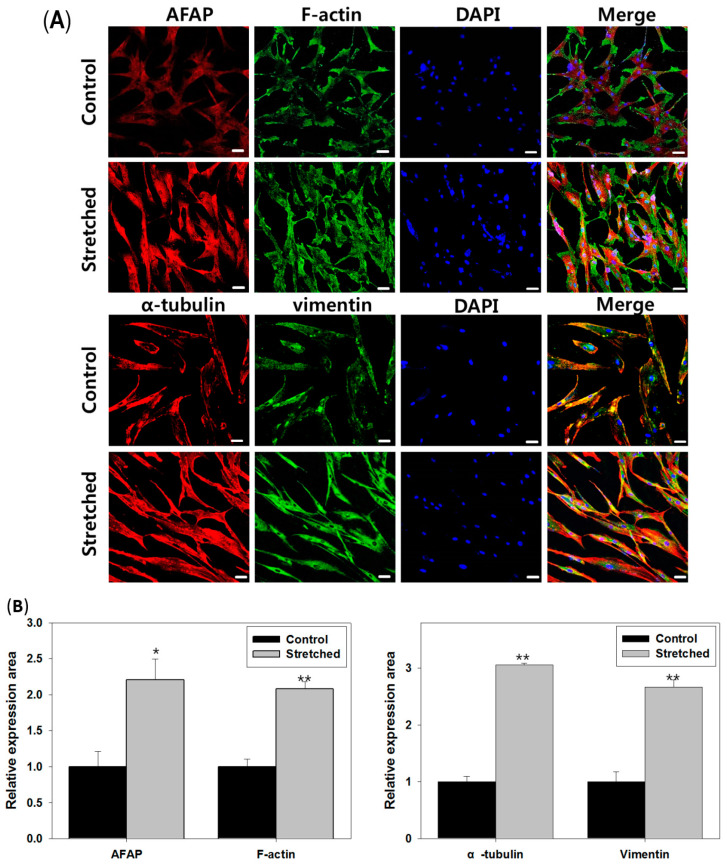
Immunohistochemical staining of KFs demonstrated an increase and change in cytoskeletal composition due to cyclic mechanical stretch. (**A**) Immunofluorescent staining images of protein markers involved in actin filament formation after being exposed to mechanical tension (×20 magnification, scale bar = 50 μm); (**B**) after experiencing mechanical tension, expression of the actin filament formation-related proteins increased, and the cells were regularly arranged. We used KFs at passage 6. All experiments were repeated three times. Error bars, s.e.m. ** *p* < 0.005, * *p* < 0.05; Student’s *t*-test.

To elucidate the underlying mechanism of increased cell proliferation and morphologic changes in fibroblasts, we performed immunofluorescent staining of the following key cytoskeletal components: intermediate filaments, microtubules, and actin filaments. KFs were subject to mechanical strain as described in previous experiments (Figure 2 and Figure 3). In response to cyclic stretch, each cytoskeletal component of the fibroblast cytoskeleton contributed to the increased thickness of the fibroblasts, which became more dispersed throughout the cell structure. The results were quantified to compare the relative expression area after mechanical strain; following mechanical stretch, each cytoskeletal component showed a significantly increased expression (** *p* < 0.005, * *p* < 0.05; Figure 5A,B). 

## 4. Discussion

In this study, we exposed cultured fibroblasts to coupled dynamic mechanical stimulation and evaluated the shear stress-dependent changes in expression of key molecules, along with phenotypic alterations. Increased cell proliferation and expression of fibrosis-related markers were noted in response to mechanical stretch, and KFs were found to be more susceptible than HDFs. Subsequent molecular analysis demonstrated an increase in TGF-β-mediated ECM deposition after cyclic stretch. Proteomic analyses revealed the upregulated expression of proteins involved in oxidative stress, and downregulation of cellular metabolism, including processes such as cell cycle regulation and proliferation. The results of in vitro cyclic stretch on dermal fibroblasts demonstrated that shear stretch induced activation of the TGF-β-mediated fibrotic cascade.

Mechanosensing is a process whereby least one chemical reaction in the cell changes in response to a change in the mechanical environment [31]. Under external mechanical stress, cells respond according to their unique mechanosensitivity profile [32]; stretched fibroblasts showed faster a proliferation rate, while their cellular structure demonstrated a large, sheet-like morphology with shorter dendritic processes than their upstretched counterparts [19,33]. New platforms have been suggested to discover molecular pathogeneses in various systemic disease models involving tissue fibrosis upon mechanical tension [30]. Evolving from the PDMS-based cell culture system used in this study, microfluidic devices have been increasingly adapted to reproduce the cyclic mechanical environment, with successful Lab-On-A-Chip models of cardiac [34] and pulmonary fibroblasts [35].

Regardless of its etiology, the fibrotic process itself is characterized by altered epithelial–mesenchymal cell interactions, inflammation, and proliferation of fibroblasts exhibiting the phenotype of myofibroblasts [3,36,37,38]. Myofibroblasts play a key role in connective tissue remodeling and reconstruction by secreting and organizing the ECM, as well as endowing the tissue with contractile forces [38,39,40]. Myofibroblasts exhibit increased responsiveness to biomechanical stimuli, which leads to further activation of fibrogenic signals [37,41,42]; persistence of fibroblast activity is mediated by reduced sensitivity to apoptotic cues and upregulated TGF-β [43]. This study demonstrated the upregulation of TGF-β and COL1A1 upon mechanical stimulation, while the downregulation of PPP2R5D was noted, suggesting a decreased sensitivity to apoptosis in KFs. Previous studies have shown increases in ROS in airway and alveolar epithelial cells in response to cyclic stretch [44]. As in systemic diseases, hypoxia is an indispensable extracellular signal that initiates the fibrotic process after tissue injury [45]. The TGF-β/Smad3 signaling pathway, crucial for the fibrotic transformation of HDFs and KFs, is also responsible for transition to the myofibroblast-like phenotype under hypoxic conditions [44,46]. Keloid tissue exhibits a hypoxic status and increased expression of hypoxic inducible factor 1α (HIF-1α), a critical mediator of hypoxia, due to microvessel occlusion. In our study, SOD2 and AKR1B1, the proteins involved in ROS scavenging, showed significant increases in response to mechanical stretch; AKR1B1 expression is related to hypoxia-driven HIF-1α signaling [47]. Positive feedback between HIF-1α and SOD2 expression levels has been reported in the context of the cellular response to hypoxia adaptation [48]. Moreover, an association between hypoxia and mechanosensitivity is depicted by recent findings regarding transient receptor potential vanilloid (TRPV4) channels, which are activated in response to changes in matrix stiffness in fibrotic diseases in a TGF-β-dependent manner [49,50]. TRPV4 channels have been particularly well studied in pulmonary and cardiac fibrosis models by mediating hypoxia-induced myofibroblast proliferation as a mechanosensitive receptor [51,52]. 

The cytoskeleton, a highly conserved feature of eukaryotic cells, crucial in the maintenance of cell shape and structure, consists of intermediate filaments, microtubules, and actin filaments, with cellular diversity in the protein components [53]. In systemic diseases, cytoskeletal dysfunction may lead to cell stiffness and fibrosis [54]; it is involved in cell motility and metabolism, as well as mechanotransductory signaling processes [55,56]. Actin acts as a pendulum, modulating the mechanoresponsiveness in a network of cytoskeleton-regulated signaling molecules. In response to mechanical stretch, fibroblasts form focal adhesions, linking actin filaments and the plasma membrane to the underlying ECM [6]. The present assay of the cytoskeletal components of KFs after mechanical stretch showed increased levels of AFAP and F-actin expression; this is consistent with a previous study reporting that mechanical stretch-induced deformation of the cytoskeleton involves activation of an AFAP-induced actin biochemical signal cascade via Src kinase activation [57]. Src modulates cell–cell contact, cell motility, and invasion of tumor cells, while promoting fibroblast activation in systemic fibrotic diseases by integrating multiple fibrogenic signal inputs [58,59,60]. In particular, phosphorylation sites of Src kinases share targets of the Raf/MEK/ERK cascade [61], which is known to promote fibrosis in keloid pathogenesis [62,63]. Therefore, the concomitant increase of AFAP and F-actin can be linked to the possible activation of a positive signaling feedback loop, which may explain the aggressiveness of KFs and their clinical tendency to expand beyond the original margin. Both in vitro and in vivo, cells are usually biochemically and mechanically supported by the ECM [64]; stiffness of the substrate can, therefore, stimulate the differentiation of fibroblasts into myofibroblasts. A dynamic signaling feedback loop after active TGF-β release leads to a change in cell phenotype. Compared with the steady state, this dynamic change in substrate stiffness affects the activation signal of the feedback loop, which corresponds with the conversion of fibroblasts to myofibroblasts [31]. Our experimental results showed that KFs respond more sensitively to mechanical stretch compared to HDFs. This finding is consistent with recent studies, which have reported that differences in tissue stiffness serve as a stimulus for tissue invasion in keloid pathogenesis, leading to a fibroproliferative phenotype [65]. In keloid tissue, this invasive and proliferative phenotype is driven by the imbalance between the increased ECM stiffness, due to elevated type I collagen, and the surrounding normal skin [66]. Mechanobiology and mechanosensing are thus emerging methods for manipulating the levels of stiffness and tension in preclinical models of fibrosis [6,64].

## 5. Conclusions

In summary, we developed a mechanically stretchable platform that enables consistent mechanical stretching of fibroblasts in a physiological cyclic stretching condition. Through proteomic analysis, we identified the target molecules of the mechanotransductory pathway in keloid development; these were validated via analysis of the mRNA levels in cultured KFs. Our platform is advantageous for investigating the influence of shear stretch forces on cells in a more physiologically accurate condition than conventional platforms. Moreover, this new platform enables investigation of the effect of mechanical stretch on cellular architecture, as well as underlying biochemical signaling cascade. Overall, we have provided evidence suggesting that cyclic stretch is a driving force in the fibrotic process of keloids, thus validating the current hypothesis of pathogenic underlying molecular mechanisms.

## Data Availability

The original contributions presented in the study are included in the article/Supplementary Materials, further inquiries can be directed to the corresponding author.

## Supplementary Materials

The following supporting information can be downloaded at: https://www.mdpi.com/article/10.3390/biomedicines12102169/s1, Figure S1: (A) Stretch device was designed to having 12 holes on its top to apply a vacuum pressure to stretchable culture plate. Measurement of dimensions seen from above. (B,C) Measurement from lateral sides; Figure S2: (A) The mechanical strain at center (r = 0) and the average strain at the region of r ~ 50% of the PDMS chamber under a pneumatic pressure. (B) The finite-element simulation result of mechanical strain under the pneumatic pressure of 20 kPa; Figure S3: (A) Mechanical strain of the PDMS platform followed by applied pneumatic pressure; Black is experimental, and Red is simulated data. (B) The repeated pneumatic pressure and the strain response with a frequency of 1 Hz. (C) The strain response measured in 0 h and after 24 h; Figure S4: Cell proliferation of HDFs (A) and KFs (B) after mechanical tension on days 1, 3, and 7. Mechanical pressure was applied for 1 h per day at 37 °C and 5% CO_2_ for up to 7 days and measured cell proliferation using the WST-1 assay; Figure S5: The morphology of HDFs (A) and KFs (B) under mechanical strain in 0, 24, and 48 h; Figure S6: The original western blotting images of HDFs and KFs; Table S1: Information of keloid fibroblast samples; Table S2: Upregulated proteins identified using the TMT labeling method; Table S3: Downregulated proteins identified using the TMT labeling method.
