# Supplementary material for: Increased Susceptibility to Mechanical Stretch Drives the Persistence of Keloid Fibroblasts: An Investigation Using a Stretchable PDMS Platform
Source: Biomedicines. 2024 Sep 24;12(10):2169. doi: 10.3390/biomedicines12102169 (PMC11504861; doi:10.3390/biomedicines12102169)
Supplement: Supplementary file 1 [file biomedicines-12-02169-s001.zip › biomedicines-3103184-supplementary.pdf]

Supplementary Figures

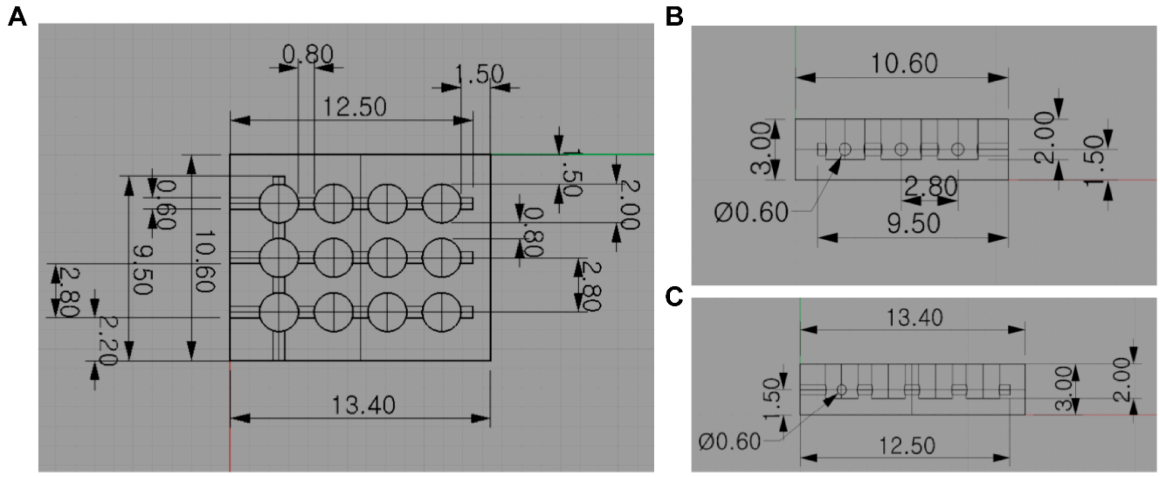

Figure S1. (**A**) Stretch device was designed to having 12 holes on its top to apply a vacuum pressure to stretchable culture plate. Measurement of dimensions seen from above. (**B,C**) Measurement from lateral sides.

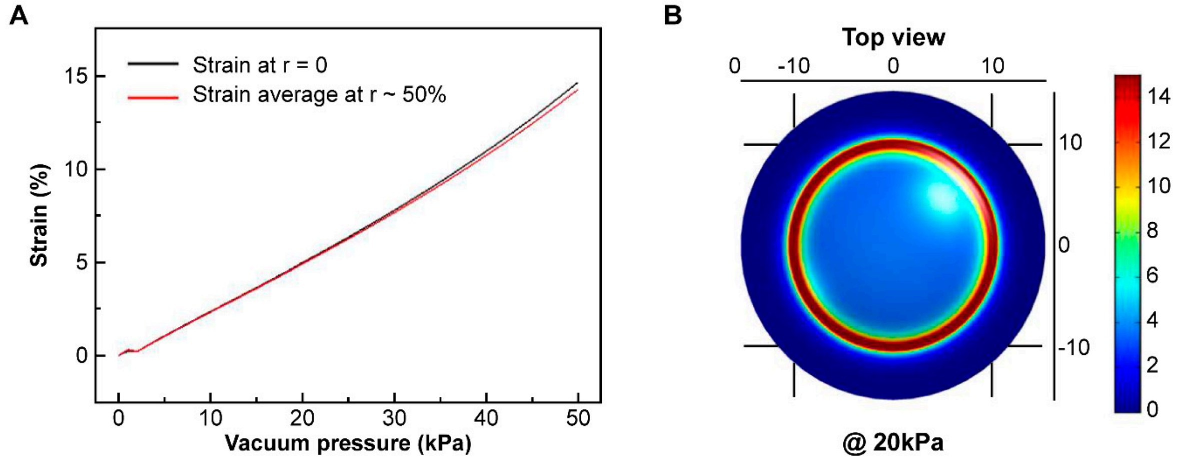

Figure S2. (**A**) The mechanical strain at center (r = 0) and the average strain at the region of r ~ 50% of the PDMS chamber under a pneumatic pressure. (**B**) The finite-element simulation result of mechanical strain under the pneumatic pressure of 20 kPa.

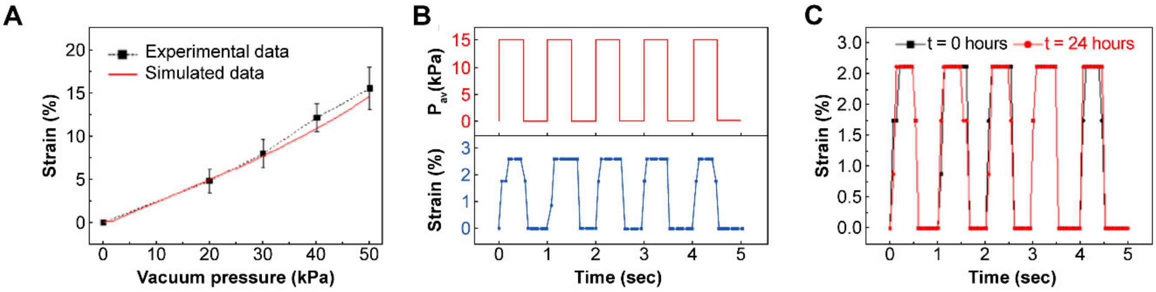

Figure S3. (**A**) Mechanical strain of the PDMS platform followed by applied pneumatic pressure; Black is experimental, and Red is simulated data. **(B)** The repeated pneumatic pressure and the strain response with a frequency of 1 Hz. **(C)** The strain response measured in 0 h and after 24 h.

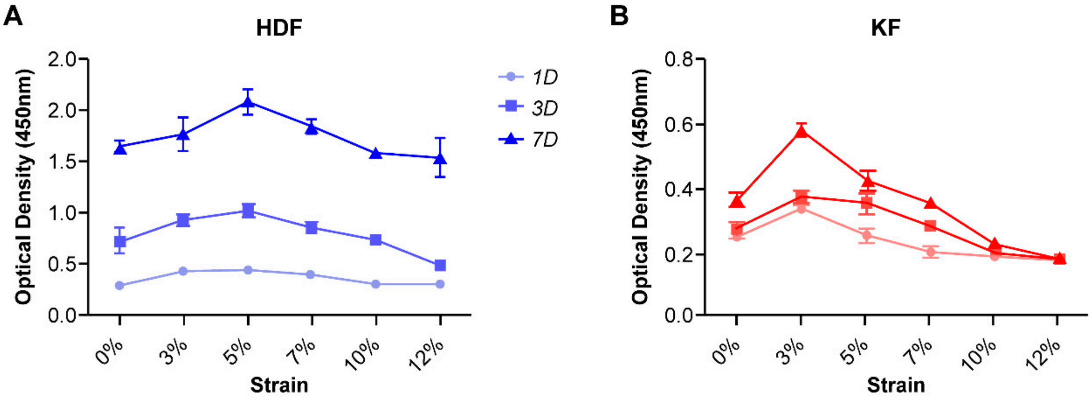

Figure S4. Cell proliferation of HDFs (**A**) and KFs (**B**) after mechanical tension on days 1, 3, and 7. Mechanical pressure was applied for 1 h per day at 37 °C and 5% CO_2 for up to 7 days and measured cell proliferation using the WST-1 assay.

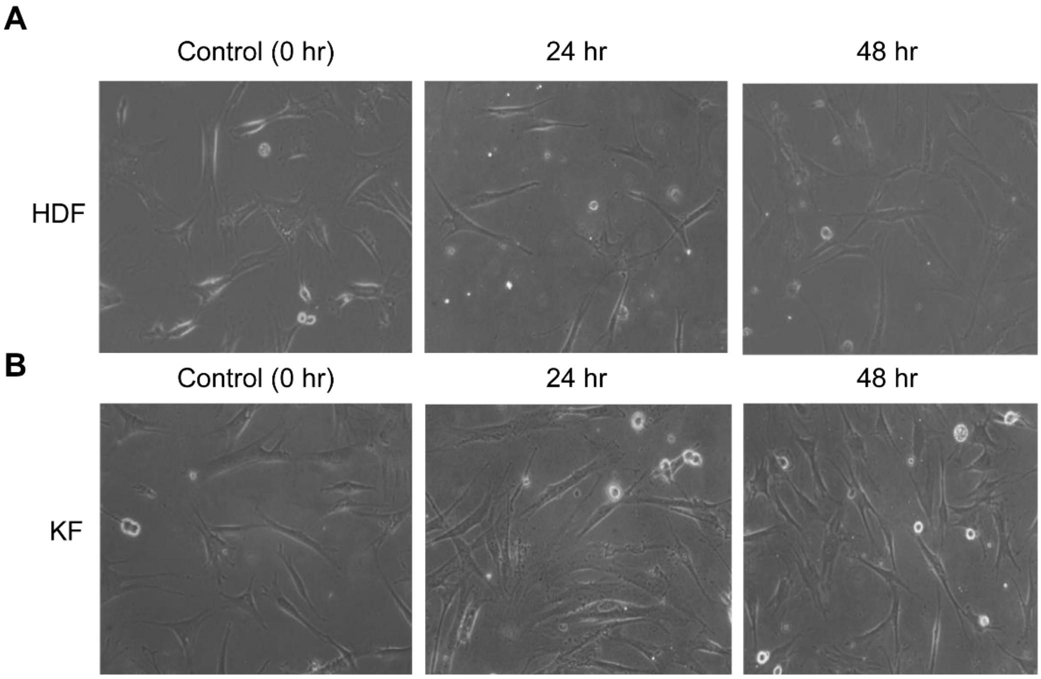

Figure S5. The morphology of HDFs (**A**) and KFs (**B**) under mechanical strain in 0, 24, and 48 h. Scale bar: 100 μm.

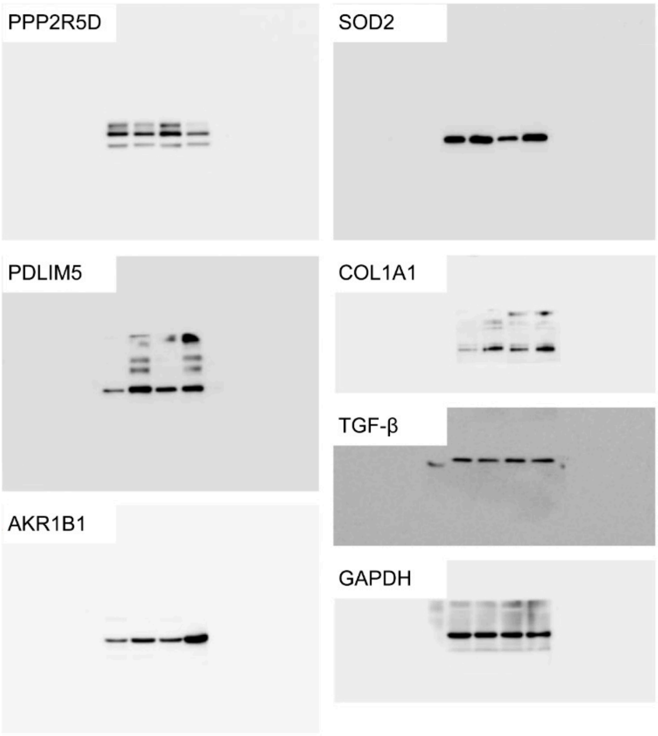

Figure S6. The original western blotting images of HDFs and KFs.

Supplementary Tables

Table S1. Information of keloid fibroblast samples.

	Sex	Age (year)	Site
KF 1	F	22	Earlobe
KF 2	F	29	Chest
KF 3	M	26	Earlobe
KF 4	F	53	Earlobe
KF 5	M	35	Chest

KF, keloid fibroblasts

Table S2. Upregulated proteins identified using the TMT labeling method.

NO	LOCUS	DESCRIPTION	ratio log2 (stretched: nonstretched)
1	sp|Q9HB40|RISC_HUMAN	SCPEP1 Retinoid-inducible serine carboxypeptidase	0.41
2	sp|Q05682-5|CALD1_HUMAN	CALD1 Isoform 5 of Caldesmon	0.41
3	sp|Q94925|GLSK_HUMAN	GLS Glutaminase kidney isoform, mitochondrial	0.41
4	sp|P42765|THIM_HUMAN	ACAA2 3-ketoacyl-CoA thiolase, mitochondrial	0.41
5	sp|P02788|TRFL_HUMAN	LTF Lactotransferrin	0.47
6	sp|O95394|AGM1_HUMAN	PGM3 Phosphoacetylglucosamine mutase	0.47
7	sp|O60486|PLXC1_HUMAN	PLXNC1 Plexin-C1	0.48
8	sp|P48163|MAOX_HUMAN	ME1 NADP-dependent malic enzyme	0.51
9	sp|P02765|FETUA_HUMAN	AHSG Alpha-2-HS-glycoprotein	0.54
10	sp|P17301|ITA2_HUMAN	ITGA2 Integrin alpha-2	0.54
11	sp|P01023|A2MG_HUMAN	A2M Alpha-2-macroglobulin	0.55
12	tr|C9JFR7|C9JFR7_HUMAN	CYC Cytochrome c (Fragment)	0.56
13	tr|K7EQ73|K7EQ73_HUMAN	DNAJC7 DnaJ homolog subfamily C member 7 (Fragment)	0.58
14	tr|F5HTY0|F5HTY0_HUMAN	PDLIM5 PDZ and LIM domain protein 5	0.58
15	sp|P15121|ALDR_HUMAN	AKR1B1 Aldose reductase	0.74
16	sp|P04179-4|SODM_HUMAN	SOD2 Isoform 4 of Superoxide dismutase	0.76

Table S3. Downregulated proteins identified using the TMT labeling method.

NO	LOCUS	DESCRIPTION	ratio log2 (stretched: nonstretched)
**1**	sp|P02452|CO1A1_HUMAN	COL1A1 Collagen alpha-1(I) chain	-0.59
**2**	sp|P07093-3|GDN_HUMAN	SERPINE2 Isoform 3 of Glia-derived nexin	-0.58
**3**	sp|Q99715|COCA1_HUMAN	COL12A1 Collagen alpha-1(XII) chain	-0.55
**4**	sp|Q5JRA6|MIA3_HUMAN	MIA3 Melanoma inhibitory activity protein 3	-0.54
**5**	sp|P12110|CO6A2_HUMAN	COL6A2 Collagen alpha-2(VI) chain	-0.51
**6**	tr|A0A087X0S5|A0A087X0S5_HUMAN	COL6A1 Collagen alpha-1(VI) chain	-0.49
**7**	sp|Q9ULD0-3|OGDHL_HUMAN	OGDHL Isoform 3 of 2-oxoglutarate dehydrogenase-like, mitochondrial	-0.47
**8**	sp|Q52LR7|EPC2_HUMAN	EPC2 Enhancer of polycomb homolog 2	-0.45
**9**	tr|A0A087WTA8|A0A087WTA8_HUMAN	COL1A2 Collagen alpha-2(I) chain	-0.45
**10**	sp|Q8NB09|SPATA5_HUMAN	SPATA5 Spermatogenesis-associated protein 5	-0.42
**11**	tr|H0Y8C4|H0Y8C4_HUMAN	PPP2R5D Serine/threonine-protein phosphatase 2A 56 kDa regulatory subunit delta isoform (Fragment)	-0.40
